# Remarks on Nervous and Mental Disorder

**Published:** 1830-07-01

**Authors:** 


### Remarks on Nervous and Mental Disorder, with especial Reference to recent Investigations on the Subject of Insanity. By *David Uwins*, M.D. Octavo, sewed, pp. 41.

From a slight collision which lately took place between Dr. Uwins and us, entirely provoked by himself, the worthy Doctor will, no doubt, consider us as his decided enemies. We do not expect to convince him to the contrary. We had no idea, at the time of the collision, that the author of the pamphlet abovementioned, was a "mad doctor;" but we now find that Dr. Uwins has been the medical superintendant of a receptacle for weak intellects, called "the Peckham Establishment," for some years past. It is well known that the insane always take most inveterate antipathies to their dearest friends; and therefore it is probable that the atmosphere of a lunatic asylum, has infected the worthy Doctor with a prepossession against men who never injured, but always befriended him. In our last number we have endeavoured to shew that all men are mad, upon some one point—and, on this principle, we should have no qualms of conscience in consigning Dr. Uwins to his own asylum, till he had abjured the monomania of egotism. After the gentle hints which we gave Dr. Uwins respecting Gifford and the Quarterly Review, we expected that common prudence would have induced him to be silent on that subject for a short time; but no:—The article on insanity in the Quarterly is uppermost again, and one would suppose that the venerable Doctor had surpassed his century, and could only remember one idea that had engrossed his intellect in early life! Yet the Doctor is in the prime of life, and has no excuse for this everlasting tale being foisted on his readers (or rather his *reviewers*) *usque ad nauseam*. While Dr. Uwins takes great care to apprize us of his early predilection for the study of mental pathology—"a subject especially to his taste"—and his appointment to the "Peckham Establishment," he declaims in good set terms against mad doctors.

"Mad, my friends, I may be; of that it is for you to judge, in the plenitude of your Metaphysico-medico legal knowledge; but 'mad doctor' I am not. Nay, with all the feeling just expressed in favour of one particular department of medical investigation and scrutiny, I have ever been averse from the principle and practice of separating insanity, strictly so called, if indeed it can be strictly so called, from other maladies which are allied to it in nature, and differ from it only in degree. In another part of this essay I shall have to state my objection to this division more at large, and *ad rem;* suffice it for the present to intimate, that to take madness under medical cognizance, specifically, and separately, and exclusively, appears to me inconsistent with that connexion and totality in which every thing connected with the sentient system ought to be received; because such partial and excluding notions insensibly lead the mind too much into that kind of favoritism, if the expression be allowed me, which cramps the mental energies, and gives rise to that jealous feeling on the part of the public, which, as we have seen in a recent instance of notoriety, is easily fomented from a spark to a flame, by the ingenuity and ability of men who, in the exercise of their professional functions, care little what sacrifice they make of truth, or who shall be the sufferer, so that the battle be won, to the successful issue of which their earnest endeavours are directed." 6.

Dr. Uwins considers it inconsistent with the nature and objects of medical science or *art*, "to divide and subdivide any part of it in the manner proposed and practised by *some persons*."

"These divisions and separations, instead of insuring 'greatness' (according to vulgar conception and phraseology) in this or that particular, is calculated to operate the very opposite effect, viz. that of producing a *littleness and narrowness* of character; for, bating *the tact which large experience cannot fail of giving*, when reputation shall run high on this or that point, (and even this tact itself may become too mechanical, and partake, therefore, too much of empirical routine,) the wisest and most efficient physician will be the man who surveys the large subject of physiological and pathological enquiry as one connected and comprehensive whole; who rejects the notion of '*caste*,' and smiles at the popular creed of this man being great in consumption, that man being 'fine' in children's complaints; of one being an adept in female diseases, of another being a good 'mad doctor.' I hope the immediate succession of these two last items will be set down by my fair readers as purely accidental." 8.

From the last part of the quotation, it is evident that Dr. Uwins expects the perusal as well as the patronage of the fair sex for his lucubrations; but we suspect that the "march of intellect" has sent most of his fair readers many a day's journey before him on the subjects which he discusses—and that all his *cotemporary* ladies of a "certain age," have had their taste for literary composition, of the UWINIAN character, most terribly vitiated of late years.

Our readers will see, in the quotation which we have inserted, that Dr. Uwins speaks of the "littleness and narrowness" which the study of particular diseases gives to the medical character—while apparently admitting, "the tact which large experience cannot fail of giving, when reputation shall run high on this or that point." There is some inconsistency even in this position; but surely there is still more inconsistency in the following observation.

"I say, that he who is really and scientifically and radically agile in one department of the healing art, must be so in all, and that the idea of a concentrated power over one class of maladies occasioning a defective judgment in others, is quite as ideal in conceit, as that of the fabulous inventors of antiquity, who put the head of a man on the shoulders of a horse." 8.

Suppose a man had studied thoroughly all branches of his profession, but that accident had directed his attention more particularly to one—say lithotomy or lithotrity; and that reputation had given him immense experience in the favourite pursuit:—are we thence to infer that this "scientifically and *radically* agile operator must be equally agile and clever in all other departments? Baron Hourteloup and Mr. Costello have had excellent and general educations; but who would conclude that these expert operators on the stone must be equally clever in all other departments of medical science? Our worthy *confrere* and ex-warden, Mr. WAKLEY,* is one of the most expert *phlebotomists* of the age; yet a tolerable acquaintance with "MODERN BABYLON," has not enabled us to discover a single instance in which his *radical* admirers have called him in to prescribe for *themselves*. Many there be who recommend him as a plebotomist for their *neighbours*—but the deuce a one have we ever seen who has confided his own precious carcase to the care of the radical reformer!! This shews that a man may be clever in one thing, though not in all.

---

* Mr. W. has *retired* from the heavy duties of the church-wardency of St. Giles's, with the title of "EX-WARDEN of the SINK PORTS."

We congratulate the worthy author on his connexion with a lunatic asylum where "his original bias towards inquiry into the intricacies of nerve and mind became still more confirmed in the exercise of his appointed duties." The following passage will shew that Dr. Uwins has not been two or three years at the "Peckham Establishment," without making some important discoveries.

"Strange as the confession will seem to some, the division between madness and mere nervousness, which I had originally conceived to be drawn out with more than just precision, lost more and more of distinctness; madness, I have repeatedly said to myself, is an arbitrary, an odious term, and the more and further we recede from the ancient notion of attaching peculiarity to the malady, the more we cease to look upon the subjects of mental disorder as εμβροντητοι, or stamped as with the mark of Cain on their forehead, the more shall we succeed in reconciling the public to our curative attempts, and the less propriety will be perceived in setting apart a class of men for the management of madness, to whom a species of peculiar power is awarded by the selection, which neither does nor ought to exist." 11.

There is refinement for you! We are to have no more *lunatic* asylums; but only *nervous* establishments:—no more strait-waistcoats:—the ladies, whose nerves are rather tumultuous, are to be controlled by golden chains, like Zenobia with fetters of gold in the triumphal procession of Aurelian.—Even the mad doctors themselves must change their designations to the more scientific terms of Neurologists!

It appears that Dr. Uwins was preparing a large work on Insanity—we beg pardon, on "Nervous and Mental Aberration," when certain "untoward events" put to flight the train of his meditations, like the stone thrown into the tranquil lake which upset the harmony of the picture reflected on its glassy bosom.

"Banks, trees, and skies, in thick disorder run!"

"I say I became intent upon publishing a work which should not be discreditable to myself, nor without its use to the profession and the public. And on this ground I was intending to proceed quietly and slowly, when, lo! circumstances occur—investigations take place—the public mind becomes agitated—respectable practitioners are hurled from popular confidence and promised wealth into contumely and poverty—the credit of the medical profession itself becomes shaken, and those members of it who have it especially in their power, from their official situation, to assert its dignity, and maintain its right, in reference to the points in debate, seem especially to be summoned from a state of supineness to be up and active.

"———Potes hoc sub casu ducere somnos?
"Nec, quæ te circum stent deinde pericula cernis?" 13.

It is certainly very laudable in Dr. Uwins, in his official capacity, to come forward and support the dignity of the profession; but how far the present brochure, which is evidently intended for general readers, is calculated to raise the literary, scientific, and philosophical character of our brethren in the eyes of the world at large, we will not venture to determine.

Dr. U. informs the public that the first patient he confined in "Peckham Asylum," was *not* mad. There is a great advantage in making a favourable impression on our readers at the beginning—and this ingenuous confession of our author cannot fail to effect that purpose. Candour is often more prepossessing than judgment—and the force of truth is irresistible:—omnia vincit veritas. Determined not to be swept away by "the *broom* of forensic authority," Dr. Uwins attacks his legal opponents with the arms of poetry, and quotes from a volume "written by one Shakespeare," the pithy sentiment respecting "the native hue of resolution being sicklied over with the pale cast of thought," although we confess that we can see no more connexion between this passage and the diagnosis of insanity, than between "the cloud-capt towers, the gorgeous palaces, or the solemn temples" of ancient Rome and the kitchen chimney of Peckham Asylum.

GIL BLAS is next pressed into the service of our author, as illustrating the philosophy of mental derangement; and the circumstance of an eloquent prelate preaching stupid nonsense after an attack of apoplexy, is adduced as proof that insanity depends on corporeal disease. But if the preaching or writing of stupid stuff be proof of either mental derangement or corporeal disease, the Lord help us! Many of us must soon take up our abode with Dr. Uwins in the "PECKHAM ESTABLISHMENT!" The following passage would justify any two physicians to *lock up* Dr. U. himself within the walls of his own asylum—if we were to act upon *his* principles.

"The *when* and the *where*, and the manner of disjoining the deranged from the social *circle*, and the degree of injury the mind has sustained, *or is likely to feel*, from the various *modes* in which the nervous organization *is called upon* to encounter disorder, constitute, indeed, the main particulars upon which medical judgment is to be exercised when summoned to determine on questions relative to mental derangement." 22.

We can form some idea of a *circle* being *deranged*. It may be changed into the form of an oval, a square, a parallelogram, a triangle, &c. but how we are to disjoin a *deranged* from a *social* circle, is not quite so comprehensible. Neither do we understand how the "nervous organization is *called upon*" to encounter certain vicissitudes of fortune—nor who the *appellants* are in such cases. Perhaps Dr. Uwins himself can enlighten us on this point.

"He recovered, however, from it so far as to be able to compose and deliver homilies; but lo! these productions, instead of being now marked by the features of legitimate and impressive eloquence, *are turgid, and bombastical, and inane;* while the preacher still imagines them *beautiful and good.*" 21.

So we fear Dr. Uwins will find his present homilies. If they make any impression at all on the general public, they will make an impression extremely unfavourable to the republic of medicine. If non-professional readers judge of our intellectual endowments by these pompous inanities, and puerile, or rather anile egotisms, the medical profession will soon be at a low ebb! But we hope for the better. We know that the SCHOOLMASTER is at work; and that the rising generation of the profession is receiving an education, general and medical, which will correct these injurious effusions of imbecility—or neutralize their effects.

Dr. Uwins will not take the advice which we proffer—namely to *write no more*. The article in the "QUARTERLY," which he considered as the brightest effusion of his genius, and the star which was to light him on to "perpetuity of fame," has proved an ignis fatuus that has led him into numerous failures. As an author and as an editor, he has been eminently unsuccessful. As a physician, as a gentleman, in all the relations of private life, we do not believe a more worthy man exists. As a *practitioner* in medicine he has our highest respect—as a *writer*, we are not singular in estimating him as peculiarly deficient in talent—maugre the judgment of the late Mr. Gifford.

